# ADNP regulates chromatin architecture and lineage fidelity during neural differentiation

**DOI:** 10.1371/journal.pgen.1012081

**Published:** 2026-04-01

**Authors:** Phillip Wulfridge, Nathaniel Rell, John Doherty, Kuo-Chen Fang, Michelle Lee Lynskey, Kavitha Sarma

**Affiliations:** 1 Gene expression and Regulation program, The Wistar Institute, Philadelphia, Pennsylvania, United States of America; 2 Epigenetics Institute, University of Pennsylvania, Philadelphia, Pennsylvania, United States of America; 3 Cell and Molecular Biology Graduate Group, University of Pennsylvania, Philadelphia, Pennsylvania, United States of America; Iowa State University, UNITED STATES OF AMERICA

## Abstract

Transition from a pluripotent to a differentiated cell state is accompanied by significant changes in genome organization. Activity dependent neuroprotective protein (ADNP) is a chromatin regulator with critical roles in neurodevelopment and limits the genomic occupancy of CTCF, a master architectural protein in genome organization, in embryonic stem cells. However, ADNP localization, function, and relationship with CTCF in differentiated neural lineages are not well studied. Here we develop a dual degron model which allows us to acutely deplete ADNP in neural progenitor cells (NPCs). We find that ADNP depletion does not impact NPC survival in the short term, but results in a genome organization switch, which favors the formation of short-range chromatin looping interactions coinciding with CTCF accumulation. Furthermore, ADNP localizes to active gene promoters in NPCs that are unoccupied by CTCF, where it prevents over-expression of genes that are activated upon neurodifferentiation and represses those involved in commitment to other lineages. Our findings uncover CTCF-dependent as well as CTCF-independent regulatory mechanisms of ADNP in NPC-specific chromatin organization and gene expression programs that may underlie its essential function in neurodevelopment.

## Author summary

The activity-dependent neuroprotective protein (ADNP) plays an important role in brain development and has been widely studied in embryonic stem cells (ESCs). However, studying its function after stem cells differentiate into neural cells has been difficult because ESCs lacking ADNP cannot form neural progenitor cells (NPCs). To overcome this limitation, we developed a model system that allows us to selectively remove ADNP after NPCs have formed. This approach enables us to examine how ADNP functions, where it localizes in the genome, and how it interacts with CTCF, a protein that helps organize the three-dimensional structure of DNA in cells. Using this system, we found that loss of ADNP in NPCs is associated with a genome-wide shift in chromatin organization, leading to changes in local DNA interactions. In NPCs, ADNP is enriched at a new set of genes, where it helps fine-tune the activity of genes that promote neurodifferentiation while repressing genes involved in alternative cell fates. Our findings suggest that ADNP acts through both CTCF-dependent and CTCF-independent mechanisms to regulate chromatin architecture in NPCs and support proper neural development.

## Introduction

Genome organization undergoes significant transformations during development, playing a critical role in ensuring proper gene expression and cell fate determination [1–4]. During development, the genome is restructured in three-dimensional space to facilitate or inhibit the interaction of regulatory elements with target genes [5]. Epigenetic modifications, such as DNA methylation and histone modifications, further influence these changes, establishing cell-type-specific gene expression patterns. Key architectural proteins, including CTCF and cohesin, help maintain these organizational frameworks while responding to developmental cues [6,7]. Disruptions in any one of these processes can lead to developmental abnormalities and disease.

The mammalian genome is folded across many scales such as A/B compartments [8,9], topologically associated domains (TADs) [10–12], sub-TADs [13], and loops [9]. CTCF is a highly conserved zinc finger protein which recognizes the CCCTC motif and its surrounding DNA sequence to bind specific genomic regions [14,15]. CTCF protein is enriched at TAD boundaries and functions to block loop extrusion by the cohesin complex [16–18]. CTCF depletion disrupts many TADs and loops, highlighting its fundamental role in genome folding [19]. CTCF binding to its consensus DNA motif can be modulated by DNA methylation [20–22] or by the binding of neighboring proteins that obscure its recognition sequence [23,24]. These mechanisms allow for dynamic regulation of CTCF localization during development, influenced by changes in DNA methylation patterns or the lineage-specific expression of competing transcription factors. As a result, CTCF occupancy—and consequently, genome organization—can be altered to fine-tune gene expression in a context-dependent manner.

Activity dependent neuroprotective protein (ADNP) is a zinc finger and homeodomain containing protein that interacts with the CHD4 chromatin remodeler and heterochromatin protein 1 (HP1) to form the ChAHP complex [25]. Mutations in the ADNP gene cause ADNP syndrome which presents as a neurodevelopmental disorder and is one of the more common genetic causes of autism spectrum disorder (ASD) [26]. On a cellular level, ADNP is crucial for mouse embryonic stem cells (mESCs) to differentiate into neural progenitor cells (NPCs) [25,27,28]. One mechanism for defective neurodifferentiation caused by ADNP loss occurs through deregulation of the Wnt signaling pathway, which is essential for neurodevelopment [27]. More recently, a conditional knockout mouse model where ADNP was specifically deleted in the dorsal telencephalon revealed a role for ADNP in the generation of upper layer neurons through the regulation of self-renewal and proliferation of apical progenitors [29].

In mESCs, ADNP as part of the ChAHP complex localizes to many genomic regions that contain B2 short interspersed nuclear elements (SINE) and that are also occupied by CTCF [23,30]. ADNP deletion in mESCs results in increased CTCF binding at a subset of these genomic sites [23,31]. In these studies, while ADNP loss did not lead to large-scale changes in genome organization, CTCF accumulation at previously ChAHP-bound sites was associated with altered boundary strength at a small number of TADs and changes in chromatin loop formation, suggesting that the ChAHP complex can regulate looping interactions by modulating CTCF occupancy. Although these studies have begun to reveal the molecular functions of ADNP in mESCs, it remains unclear whether these are preserved during neural lineage commitment, or if ADNP plays additional or alternative roles in differentiated cells. However, studying the consequences of ADNP loss in NPCs is particularly challenging because ADNP knockout mESCs do not survive the early stages of neurodifferentiation [25,28]. As a result, a thorough analysis of ADNP molecular interactions and functions in the neural lineage has remained difficult.

Here, we developed an ADNP degron model that allows us to deplete ADNP in NPCs without affecting their survival. Using this model, we explore ADNP localization and molecular function in NPCs and identify contributions to lineage-specific genome organization and gene expression programs.

## Results

### ADNP and CTCF redistribute during neurodifferentiation

**Fig 1:**
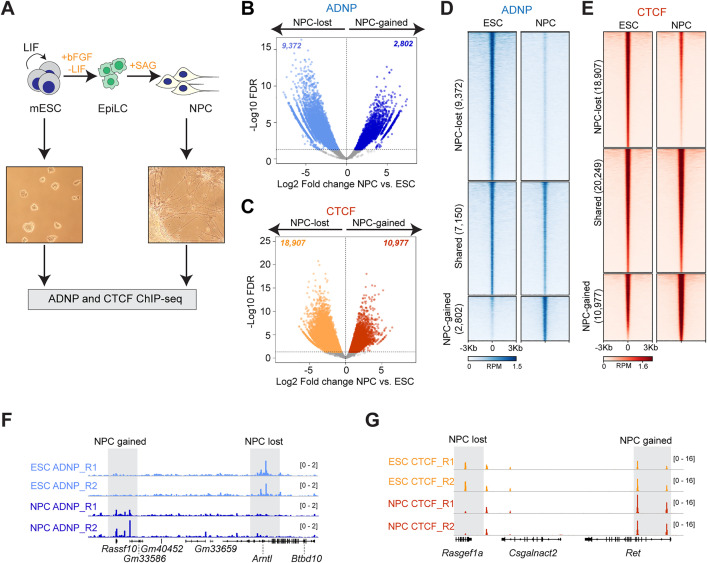
ADNP and CTCF are redistributed genome-wide upon neurodifferentiation. **(A)** Experimental schematic of neural differentiation and ChIP-seq. LIF and GSK/MEK inhibitors are withdrawn from mESCs and basic fibroblast growth factor (bFGF) is added, resulting in the generation of EpiLCs. Addition of smoothened agonist (SAG) to EpiLCs results in formation of neural progenitor cells (NPCs). Representative images of mESC and NPC stages are shown. Cells from mESC and NPC stages are processed for ADNP and CTCF ChIP-Seq. **(B)** Volcano plot showing log2 fold changes in ADNP binding between mESC and NPC stages on the x-axis and -log10 FDR on the y-axis. 2,802 NPC-gained (dark blue) and 9,372 NPC-lost (light blue) ADNP sites are indicated passing the 0.05 FDR cutoff for significance. Volcano plot showing log2 fold changes in CTCF binding between mESC and NPC stages on the x-axis and -log10 FDR on the y-axis. 10,977 NPC-gained (red) and 18,907 NPC-lost (orange) CTCF sites are indicated passing the 0.05 FDR cutoff for significance. **(D)** Heatmap of ADNP ChIP-Seq signal in mESC and NPC stages across NPC-lost (9,372), shared (7,105), and NPC-gained (2,802) ADNP peaks. Rows within each group are sorted in decreasing order by mean signal across all samples. RPM, reads per million. **(E)** Heatmap of CTCF ChIP-Seq signal in mESC and NPC stages across NPC-lost (18,907), shared (20,249), and NPC-gained (10,977) ADNP peaks. Rows within each group are sorted in decreasing order by mean signal across all samples. RPM, reads per million. **(F)** Genome browser view of a region of chromosome 7 showing ADNP ChIP-seq signal (reads per million, RPM) in 2 independent mESC and NPC replicates. NPC-gained and NPC-lost ADNP peaks are highlighted. **(G)** Genome browser view of a region of chromosome 6 showing CTCF ChIP-seq signal (reads per million, RPM) in 2 independent mESC and NPC replicates. NPC-gained and NPC-lost CTCF peaks are highlighted.

ADNP and CTCF co-localize in mESCs [23], raising the question of whether this remains true upon neurodifferentiation. To answer this, we differentiated mESCs into NPCs using a two-step strategy via an epiblast-like cell (EpiLC) intermediate and performed ChIP-seq for ADNP and CTCF to profile their genomic occupancy (Fig 1A). We identified 14,232 ADNP peaks in mESCs and 5,883 in NPCs across two replicates. We also found 46,046 CTCF peaks in mESCs and 30,140 peaks in NPCs. We examined mESC CTCF signal across the 14,232 mESC ADNP peaks and observed that these peaks are divided into a subset with little CTCF signal and a subset with markedly higher CTCF occupancy, consistent with previous reports of partial co-localization between these two factors (S1A Fig, blue). Interestingly, when we examined NPC CTCF signal across 5,883 NPC ADNP peaks, we found a lower proportion of sites with high CTCF occupancy (S1A Fig, red), suggesting that ADNP-CTCF co-localization may become weaker upon differentiation.

To more closely determine how ADNP and CTCF are redistributed genome-wide upon neurodifferentiation, we identified differentially bound regions in NPCs as compared to mESCs. Our analysis revealed widespread changes where both ADNP and CTCF were lost from specific sites in mESCs and are either retained or gained at other sites in NPCs (Fig 1B and 1C). We identified 9,372 regions where ADNP is present in ESCs but lost upon differentiation to NPCs, as well as 7,150 regions that retain ADNP. Conversely, 2,802 regions were nearly devoid of ADNP signal in ESCs and gained ADNP binding in NPCs (Fig 1D). Similar analysis of differentially bound CTCF sites identified 18,907 regions that are lost upon differentiation of ESC to NPC, 20,249 that are retained, and 10,977 sites that are gained (Fig 1C and 1E). Notably, “NPC-gained” CTCF sites are bound by CTCF even in mESCs, and CTCF binding strengthens further in NPCs (S1B Fig, right panel). In contrast, sites that gain ADNP in NPCs show very low levels of ADNP in the undifferentiated state, suggesting that ADNP is recruited *de novo* to these sites upon neurodifferentiation (S1C Fig, right panel). For example, the *Rassf10* and *Gm33586* genes are devoid of ADNP signal in ESC but show clear ADNP presence in NPCs (Fig 1F). At the *Arntl* gene, ADNP is present in ESCs, but lost upon neurodifferentiation (Fig 1F). In the case of CTCF, the *Rasgef1a* gene loses CTCF enrichment in NPC, while two sites within the *Ret* gene already contain CTCF in ESCs and show further increase in binding in NPCs (Fig 1G).

ADNP loss results in gained CTCF at a subset of binding sites in mESCs [23], suggesting that these two factors may compete for binding. Thus, one possibility for ADNP redistribution in NPCs is that it localizes *de novo* to sites previously occupied by CTCF in mESCs. To determine whether ADNP is gained in NPC at sites that correspond with CTCF loss, we examined CTCF signal at NPC-gained ADNP sites. We found that CTCF occupancy at these ADNP peaks was very low in both the ESC and NPC stage, compared to CTCF peaks that are characterized by much higher signal (S1D and S1E Fig). Similarly, regions that gained CTCF in NPC did not show appreciable changes in ADNP levels between ESCs and NPCs (S1F and S1G Fig), and regions that lose CTCF in NPCs do not gain ADNP (S1G Fig), suggesting that CTCF and ADNP redistribution in NPCs is not driven by competition for binding sites, but by other mechanisms of active accumulation. We also found that NPC-gained ADNP sites have low CTCF signal compared to NPC-lost ADNP sites, or shared ADNP sites which are enriched for CTCF in both ESC and NPC stages (S1E Fig), suggesting that this subset of NPC-specific ADNP binding events may have CTCF-independent regulatory roles.

### ADNP is gained at active promoters upon neurodifferentiation

**Fig 2:**
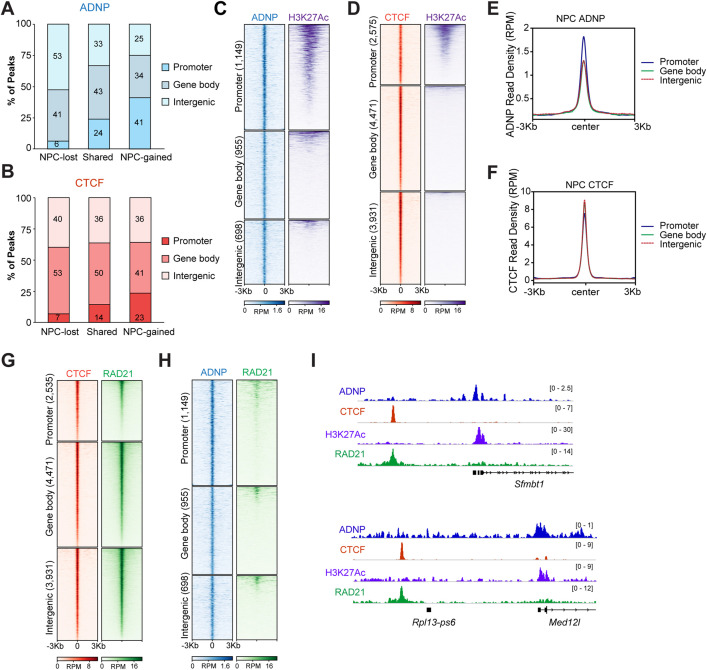
ADNP is predominantly gained at active promoters and CTCF at cohesin binding sites in NPCs. **(A)** Bar chart displaying distribution of NPC-lost, shared, and NPC-gained ADNP sites across promoter, gene body, and intergenic genomic features. **(B)** Bar chart displaying distribution of NPC-lost, shared, and NPC-gained CTCF sites across promoter, gene body, and intergenic genomic features. **(C)** Heatmap of ADNP and H3K27Ac ChIP-seq signal in NPCs across 2,802 NPC-gained ADNP sites subdivided by their distribution over promoter (1,149), gene body (955), or intergenic (698) regions. Rows within each group are sorted in decreasing order by mean signal across all samples. RPM, reads per million. **(D)** Heatmap of CTCF and H3K27Ac ChIP-seq signal in NPCs across 10,977 NPC-gained CTCF sites subdivided by their distribution over promoter (2,535), gene body (4,471), or intergenic (3,931) regions. Rows within each group are sorted in decreasing order by mean signal across all samples. RPM, reads per million. **(E)** Signal plot of ADNP ChIP-seq signal across all NPC-gained ADNP peaks subdivided by their distribution over promoter, gene body, or intergenic regions. RPM, reads per million. **(F)** Signal plot of CTCF ChIP-seq signal across all NPC-gained CTCF peaks subdivided by their distribution over promoter, gene body, or intergenic regions. RPM, reads per million. **(G)** Heatmap of CTCF and RAD21 ChIP-seq signal in NPCs across 10,977 NPC-gained CTCF sites subdivided by their distribution over promoter (2,535), gene body (4,471), or intergenic (3,931) regions. Rows within each group are sorted in decreasing order by mean signal across all samples. RPM, reads per million. **(H)** Heatmap of ADNP and RAD21 ChIP-seq signal in NPCs across 2,802 NPC-gained ADNP sites subdivided by their distribution over promoter (1,149), gene body (955), or intergenic (698) regions. Rows within each group are sorted in decreasing order by mean signal across all samples. RPM, reads per million. **(I)** Genome browser views of the *Sfmbt1*, *Rpl13-ps6*, and *Med12l* genes showing ADNP, CTCF, H3K27Ac, and RAD21 ChIP-seq signal (reads per million, RPM) in NPCs.

Next, we asked if ADNP and CTCF redistribute to different genomic features during ESC-to-NPC differentiation. Analysis of ADNP binding sites that are present in ESCs and lost in NPCs revealed that these sites are predominantly intergenic or within gene bodies (Fig 2A). In contrast, almost half of NPC-gained ADNP sites are at gene promoters (Fig 2A). A similar analysis of CTCF showed that, like ADNP, CTCF localization undergoes a mild shift from intergenic sites and gene bodies towards gene promoters at the NPC stage (Fig 2B), although the enrichment of NPC-gained CTCF at promoters is not as pronounced as with NPC-gained ADNP. Interestingly, 47% of promoter NPC-gained ADNP sites were acetylated at histone H3 lysine 27 (H3K27ac), indicating active transcription (Fig 2C and 2I). CTCF also localized to active promoters in NPCs, but to a lesser extent than ADNP (40%, Fig 2D). The magnitude of ADNP and CTCF enrichment also differed across promoters, gene bodies, and intergenic sites. In NPCs, ADNP was present at highest levels at promoters (Fig 2C and 2E), while CTCF showed the highest accumulation within gene bodies and intergenic sites (Fig 2D and 2F).

CTCF functions to limit loop extrusion by cohesin, a protein complex consisting of SMC1, SMC3, and RAD21 [9,18]. The partnership between CTCF and cohesin in genome organization is evident through their co-localization at genomic regions, many of which serve as loop anchors. We found that most NPC gained CTCF sites show strong enrichment for the RAD21 component of the cohesin complex, which is most pronounced across intergenic and gene body peaks (Fig 2G and 2I). Notably, no such relationship was observed between ADNP and RAD21 (Fig 2H and 2I). Our results point to CTCF- and cohesin-independent roles for ADNP that may be unrelated to genome organization in the neural lineage.

### ADNP localizes to MIR elements in the neural lineage

CTCF and ADNP are enriched at SINE B2 repeats in mESCs [23] and ADNP also localizes to Alu repeats [32], the most abundant SINE family repeat in humans. We confirmed that both ADNP and CTCF are enriched at SINE B2 elements in mESCs compared to background (S2A and S2D Fig, left). Sites bound by ADNP or CTCF in both mESCs and NPCs also overlap with SINE B2 elements (S2B and S2E Fig). Interestingly, we found that upon neurodifferentiation, NPC-gained ADNP—and to a lesser extent, NPC-gained CTCF—shows increased enrichment at a different class of SINE elements known as Mammalian-wide Interspersed Repeats (MIRs) (S2C and S2F Fig), which are associated with both enhancer and insulator functions [33]. NPC-gained ADNP sites have by far the highest enrichment for these elements, with 14.7% of these sites overlapping a MIR element compared to only 2.3% of random genomic regions (S2G Fig). Interestingly, MIR elements were previously reported as enriched in tissue-specific enhancers [34], suggesting that ADNP may have lineage-specific regulatory function and leading us to examine ADNP’s roles in regulating gene expression in the neural lineage.

### Development of a dual-degron system for acute depletion of ADNP in the neural lineage

ADNP is critical for the process of neurodifferentiation [25,27,28]. Mouse ESCs deficient for ADNP die during the initial stages of differentiation to NPCs. Conditional knockout mouse models have provided important insight into ADNP functions in specific brain regions [29], but, because of cellular heterogeneity, they are not ideal for deep mechanistic studies in specific cell types. To study ADNP molecular functions in the neural lineage, it becomes crucial to deplete ADNP *after* ESCs have successfully committed and transitioned to neural cell types such as neural progenitors. However, NPCs are notoriously difficult to transfect making knockdown studies challenging, prompting us to explore degron models to acutely deplete ADNP in NPCs. Fusion of endogenous proteins to auxin inducible degradation (AID) or FKBP12^F36V^ tags are two widely used systems for acute protein depletion [35,36]. While efficient, one limitation of this system is that addition of the FKBP12^F36V^ tag can introduce instability of tagged protein that results in lowered baseline levels of protein. We introduced the FKBP12^F36V^ and V5 tags into the 3’ end of the endogenous *Adnp* gene. In these ADNP-FKBP-V5 knock-in mESCs, we confirmed that V5 epitope tag at the C terminus of the FKBP protein is detectable in the knock-in clones, but not in parental mESCs, and is degraded upon addition of the dTAG-13 small molecule (S3A Fig, top panel). However, we also found that introduction of these tags results in spontaneous drug-independent ADNP protein degradation, possibly through protein destabilization. While untagged ADNP is clearly visible in WT mESCs, the tagged ADNP protein is almost undetectable in ADNP-FKBP-V5 knock-in mESC clones even in the absence of dTAG-13 (S3A Fig, middle panel, compare lane 1 to lanes 2 and 4). These results indicated that the FKBP-V5 tag alone was insufficient to successfully produce an ADNP degron model that is suitable for neurodifferentiation.

**Fig 3:**
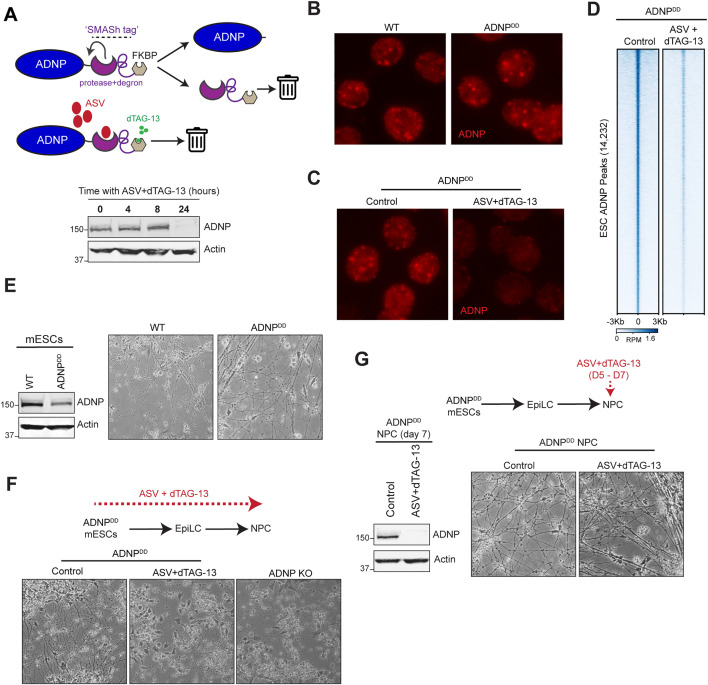
A dual SMASh-FKBP degron system for acute depletion of ADNP in NPCs. **(A)** Schematic of the ADNP-SMASh-FKBP12^F36V^ dual-degron (ADNP^DD^) system. Endogenous ADNP is tagged with both SMASh and FKBP. In the absence of drug treatment (top), the SMASh tag is self-cleaved by NS3 protease, resulting in release of the SMASh and FKBP tags and wildtype ADNP. Upon addition of asunaprevir (ASV) and dTAG-13 (middle), protease activity is inhibited, and the FKBP domain targets ADNP-SMASh-FKBP12^F36V^ for degradation. Bottom, Western blot for ADNP and actin in ADNP^DD^ mESCs after 0, 4, 8, or 24 hours of treatment with ASV and dTAG-13. Antibodies are indicated on the right. **(B)** Immunostaining of wildtype and ADNP^DD^ mESCs for ADNP (red). **(C)** Immunostaining of ADNP^DD^ mESCs for ADNP (red) before (control) and after 24hr treatment with ASV and dTAG-13. **(D)** Heatmap of ADNP ChIP-seq signal in control and drug-treated ADNP^DD^ mESCs across 14,232 mESC ADNP peaks. Rows within each group are sorted in decreasing order by mean signal across all samples. RPM, reads per million. **(E)** Left, Western blot for ADNP and actin in wildtype and ADNP^DD^ mESCs without treatment with ASV and dTAG-13. Antibodies are indicated on the right. Right, representative images of wildtype and ADNP^DD^ NPCs on day 6 of neurodifferentiation. **(F)** Top, timeline schematic of neurodifferentiation experiment in which ASV and dTAG-13 treatment is started in ADNP^DD^ mESCs prior to and throughout induction of differentiation. Bottom, representative images of control and drug-treated ADNP^DD^ cells and ADNP KO cells on day 7 of differentiation. **(G)** Top, timeline schematic of neurodifferentiation experiment in which ADNP^DD^ NPCs are treated with ASV and dTAG-13 on days 5–7 of differentiation. Bottom left, Western blot for ADNP and actin in control and drug-treated ADNP^DD^ NPCs harvested on day 7 of differentiation. Antibodies are indicated on the right. Bottom right, representative images of control and drug-treated ADNP^DD^ NPCs on day 7 of differentiation.

To address this complication, we switched to a self-excising degron system called Small Molecule-Assisted Shutoff (SMASh) for ADNP degradation (S3B Fig, schematic) [37]. The SMASh tag consists of the Hepatitis C virus (HCV) NS3 protease linked to an 8 amino-acid NS4A sequence, which improves cleavage efficiency and inhibitor binding of the NS3 protease. In the absence of the protease inhibitor asunaprevir (ASV), the SMASh tag is cleaved during translation, releasing an untagged protein that is expected to have the same stability as the endogenous version in vivo (S3B Fig). Addition of ASV inhibits NS3 protease activity and self-cleavage of the SMASh tag, which leads to degradation of the fusion protein (S3B Fig). We inserted the SMASh tag at the 3’ of endogenous *Adnp* and found that in the absence of ASV, untagged ADNP was released and was detectable at similar levels as WT ADNP in parental mESCs (S3B Fig, bottom). Addition of ASV for 24 hours resulted in ADNP degradation as evidenced by the disappearance of ADNP at its expected molecular weight. However, ASV addition also resulted in the appearance of a higher molecular band that was detectable by ADNP antibody (S3B Fig, asterisk), suggesting incomplete degradation. To degrade any residual ADNP-SMASh protein, we dual-tagged *ADNP* to produce *ADNP-SMASh-FKBP12*^*F36V*^, which, in theory, would be degraded by the simultaneous addition of ASV and dTAG-13 (Fig 3A, top). In this “ADNP-dual degron” (ADNP^DD^) mESC line, the addition of ASV and dTAG-13 resulted in near-complete depletion of ADNP within 24 hours (Fig 3A, bottom). We further characterized the ADNP^DD^ cell line to show that ADNP localized to pericentromeres as in the parental line (Fig 3B) and that treatment with ASV and dTAG-13 resulted in loss of ADNP signal from pericentromeres (Fig 3C) as well as a genome-wide loss of ADNP ChIP signal (Fig 3D). Furthermore, CTCF levels and localization are not affected by ADNP depletion (S3C and S3D Fig).

### ADNP^DD^ mESCs are competent for neurodifferentiation

Next, we tested the suitability of the ADNP^DD^ model for neurodifferentiation. We found that while ADNP protein levels were reduced in ADNP^DD^ mESCs compared to the parental ESCs (Fig 3E, left), this cell line is still able to differentiate into NPCs in the absence of ASV and dTAG-13 (Fig 3E, right). This is observed phenotypically as the production of long neurite-like extensions at the end of the differentiation protocol. We additionally performed RNA-seq in ADNP^DD^ mESCs and NPCs to confirm that neural progenitor gene expression programs are successfully activated in this cell line. Principal coordinate analysis of RNA-seq data revealed that parental ESC and ADNP^DD^ ESCs clustered together, while NPCs derived from parental or ADNP^DD^ lines clustered close together and away from ESCs, showing overall similarities between the parental and ADNP^DD^ lines (S3E Fig). Furthermore, we observed complete downregulation of mESC markers including *Oct4* and *Nanog* (S3F Fig) accompanied by upregulation of neural lineage markers such as *Sox4* and *Nes* in NPCs derived from ADNP^DD^ ESCs (S3G Fig), as well as an overall enrichment for neurodevelopmental pathways in genes upregulated in ADNP^DD^ NPCs (S3H Fig). Finally, we pre-treated ADNP^DD^ mESCs with ASV and dTAG-13 for 24 hours prior to initiation of neurodifferentiation and found that these ADNP-depleted mESCs undergo progressive cell death during the differentiation process, phenocopying the failure to form neural progenitors observed in ADNP knockout mESCs[28] (Fig 3F). Altogether, our results with this ADNP degron model reproduce and support previous findings showing that ADNP is necessary for neurodifferentiation of mESCs.

The successful generation of a model where ADNP can be depleted in the neural lineage allowed us to examine whether ADNP is important for survival at the differentiated NPC stage. To test this, we differentiated ADNP^DD^ mESCs for 5 days, then treated with ASV and dTAG-13 for 2 days. We confirmed that this 2-day treatment with ASV and dTAG-13 resulted in complete loss of ADNP in ADNP^DD^ NPCs (Fig 3G, left) Interestingly, day 7 NPCs do not show changes in viability upon ADNP loss (Fig 3G, right), suggesting that ADNP is not essential for survival once cells have transitioned to the neural progenitor stage.

### ADNP depletion in NPCs reveals CTCF-dependent regulation of genome organization

**Fig 4:**
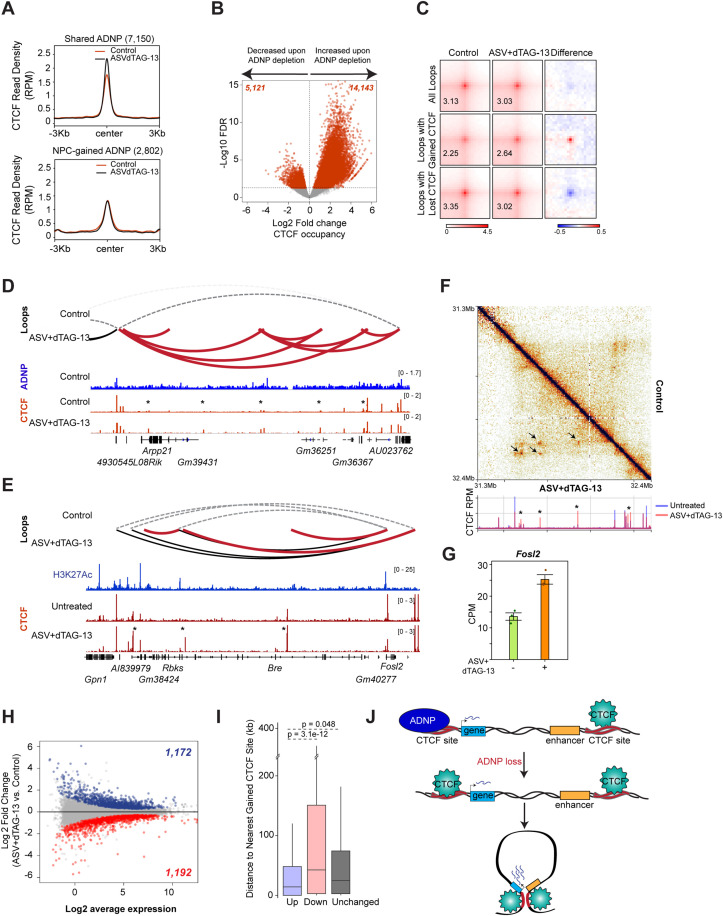
Dysregulation of CTCF binding and genome organization upon ADNP depletion in NPCs. **(A)** Signal plot of CTCF ChIP-seq signal in control and drug-treated ADNP^DD^ NPCs across 7,150 shared (top) or 2,802 NPC-gained (bottom) ADNP peaks. RPM, reads per million. **(B)** Volcano plot showing log2 fold changes in CTCF binding between control and drug-treated ADNP^DD^ NPCs on the x-axis and -log10 FDR on the y-axis. 14,143 gained sites with increased CTCF occupancy and 5,121 lost sites with decreased CTCF (red) are indicated passing the 0.05 FDR cutoff for significance. **(C)** Normalized aggregate peak analysis (APA) of 10-kb resolution HiC data comparing chromatin loop strength in control and drug-treated ADNP^DD^ NPCs across all called loops (top), loops with anchors overlapping one or more gained CTCF sites (middle), or loops with anchors overlapping one or more lost CTCF sites (bottom). Peak to lower left (P2LL) ratios are indicated in the lower left corners of APA plots. Rightmost plots display the difference in scores between control and drug-treated NPCs. **(D)** Genome browser view of a region of chromosome 9 showing called chromatin loops, ADNP ChIP-seq signal (reads per million, RPM), and CTCF ChIP-seq signal (RPM) in control and drug-treated ADNP^DD^ NPCs. Chromatin loops called in control ADNP^DD^ NPCs are shown as dashed grey lines and those conserved in drug- treated NPCs are in black. Chromatin loops specifically called in drug-treated NPCs whose anchors overlap one or more gained CTCF sites are highlighted in red and regions with CTCF gains are labeled with asterisks (*). **(E)** Genome browser view of a region of chromosome 5 showing called chromatin loops and CTCF ChIP-seq signal (reads per million, RPM) in control and drug-treated ADNP^DD^ NPCs as well as H3K27Ac ChIP-seq signal (RPM) in wildtype NPCs. Chromatin loops called in control ADNP^DD^ NPCs are shown as dashed grey lines and those conserved in drug- treated NPCs are in black. Chromatin loops called in drug-treated NPCs whose anchors overlap one or more gained CTCF sites are highlighted in red and regions with CTCF gains are labeled with asterisks (*). **(F)** 5-kb resolution Hi-C map of the region displayed in panel E, showing observed contacts in control (upper right triangle) and drug-treated (lower left triangle) ADNP^DD^ NPCs. Sites with increased contact frequency in drug-treated NPCs are indicated with arrows. CTCF ChIP-seq tracks for control and drug-treated NPCs are displayed below the map and regions with CTCF gains are labeled with asterisks (*). **(G)** Bar chart showing expression of the *Fosl2* gene in counts per million (CPM) in control and drug-treated ADNP^DD^ NPCs. Circles indicate individual biological replicates. Data are presented as mean values ± SEM. **(H)** MA plot of RNA-seq expression between control and drug-treated ADNP^DD^ NPCs. Blue and red dots indicate differentially expressed genes (adjusted P-value ≤ 0.05) that are upregulated (blue) or downregulated (red) in drug-treated NPCs compared to control. **(I)** Boxplot of distance to nearest gained CTCF site, in kb, of genes that are upregulated, downregulated, or unchanged in drug-treated ADNP^DD^ NPCs compared to control. Box, 25th percentile – median – 75th percentile. Whiskers extend to 1.5x interquartile range. p-values, Welch’s two-sided t-test. **(J)** Model of CTCF-dependent ADNP function in neurodifferentiation. In wildtype cells, ADNP binds in the vicinity of some CTCF sites to block CTCF binding. In the absence of ADNP, these sites become exposed and allow CTCF to bind, resulting in the *de novo* formation of chromatin loops that facilitate promoter-enhancer contacts for improper upregulation of genes.

Shared ADNP sites that are present in both ESCs and NPCs remain enriched for CTCF in the NPC stage (S1E Fig), suggesting that these sites retain a CTCF-related role in these cells. To further explore the relationship between shared ADNP and CTCF using our degron system, we performed CTCF ChIP in control and drug-treated ADNP^DD^ mESCs and NPCs. ADNP knockout mESCs accumulate CTCF at a subset of genomic sites [23,31]. Consistent with these previous studies, we found that acute depletion of ADNP in ADNP^DD^ mESCs results in CTCF accumulation at 1,102 sites (S4A Fig). Next, we examined CTCF behavior in NPCs after ADNP depletion and found that CTCF occupancy increases across shared ADNP peaks (Fig 4A), mirroring what is observed in mESCs and supporting that ADNP maintains a role in regulation of CTCF. Strikingly, however, CTCF binding does not increase across NPC-gained ADNP sites upon ADNP loss (Fig 4A), suggesting that these NPC-specific sites have a CTCF-independent function. Differential binding analysis found 19,264 sites that showed significant changes in CTCF binding in drug-treated NPCs compared to control. Of these, 14,143 sites gained CTCF and 5,121 sites lost CTCF upon ADNP depletion (Fig 4B), indicating a trend towards increased CTCF occupancy similar to that observed in mESCs. Most CTCF gains and losses occur at gene bodies and intergenic regions (S4B Fig). Significantly gained CTCF sites are enriched for ADNP peaks that are shared between ESC and NPC (S4C Fig) but depleted for NPC-gained ADNP peaks (S4D Fig), further consistent with the notion that shared but not NPC-gained ADNP sites play roles in CTCF regulation.

Next, we examined whether ADNP-dependent changes in CTCF at the NPC stage are associated with alterations in genome organization. ADNP knockout in mESCs was previously reported to have effects on genome organization predominantly at the level of chromatin looping, with no change in compartmentalization and little change at the TAD level, with shifts in looping linked to changes in CTCF occupancy [23]. We performed Hi-C in ADNP^DD^ NPCs with or without ASV and dTAG-13 treatment. Similar to observations in ADNP knockout mESCs, we did not detect visible alterations in compartment level organization (S4E Fig) or a significant change in TAD numbers upon ADNP depletion in NPCs (S4F Fig). Interestingly, however, the number of called loops increased (S4G Fig), and average loop size was smaller (S4H Fig), in drug-treated NPCs. Such a decrease in loop size is consistent with the loss of some long-range contacts and gain of short-range interactions, which could occur due to redistribution of CTCF to binding sites uncovered by ADNP depletion. To examine this possibility, we performed aggregate peak analysis (APA) on loops categorized by overlap of loop anchors with gained or lost CTCF sites. APA revealed that contact frequency is increased upon ADNP depletion specifically at loops whose anchors contain gained CTCF and decreased at loops where CTCF is lost (Fig 4C). Furthermore, the presence of gained CTCF sites at anchors of loops identified only in drug-treated NPCs was visually evident at many individual genomic regions (Fig 4D and 4E).

Chromatin looping can regulate gene expression by placing promoter and enhancer elements in close physical proximity. We performed RNA-Seq on ADNP^DD^ NPCs with or without ASV and dTAG-13 treatment and cross-referenced against our looping data. We found that in some instances, alterations in looping interactions were associated with expression changes of nearby genes. For example, contact frequency is strengthened for several loops connecting the promoter of *Fosl2*, an immediate-early gene in the Fos protein family that is expressed in the brain and that serves as a marker of neuronal activity [38–40], to distant upstream regions (Fig 4E and 4F). Interestingly, the other ends of these loops are enriched not only for gained CTCF, but also for H3K27Ac, suggesting that these gained interactions could position the *Fosl2* gene closer to other activated genes or enhancers. Accordingly, expression of *Fosl2* is upregulated upon ADNP depletion compared to control ADNP^DD^ NPCs (Fig 4G). This pattern can be observed at many other genes, where increases in CTCF occupancy at nearby sites and gain of looping interactions (S4I and S4J Fig) is accompanied by upregulation of expression upon ADNP depletion. Next, we asked if genes with altered expression are closer on average to gained CTCF sites, which have strengthened looping interactions upon ADNP loss (Fig 4C). We performed differential expression analysis on RNA-Seq data from control and drug-treated ADNP^DD^ NPCs to identify 1,172 genes that are significantly upregulated upon ADNP depletion compared to control NPCs and 1,192 genes that are downregulated (Fig 4H), then quantified the distance between these genes to the nearest gained CTCF site. We found that upregulated genes are significantly closer to gained CTCF sites than either downregulated genes or genes with unchanged expression (Fig 4I), while downregulated genes are farther still compared to unchanged genes. This result further supports that increased looping strength through CTCF occupancy upon ADNP loss is accompanied by increased expression of proximal genes (Fig 4J). Our results suggest that shared ADNP sites regulate gene expression during neurodifferentiation by limiting CTCF occupancy and preventing formation of spurious looping interactions.

### ADNP represses mesoderm cell fate commitment genes in NPCs

**Fig 5:**
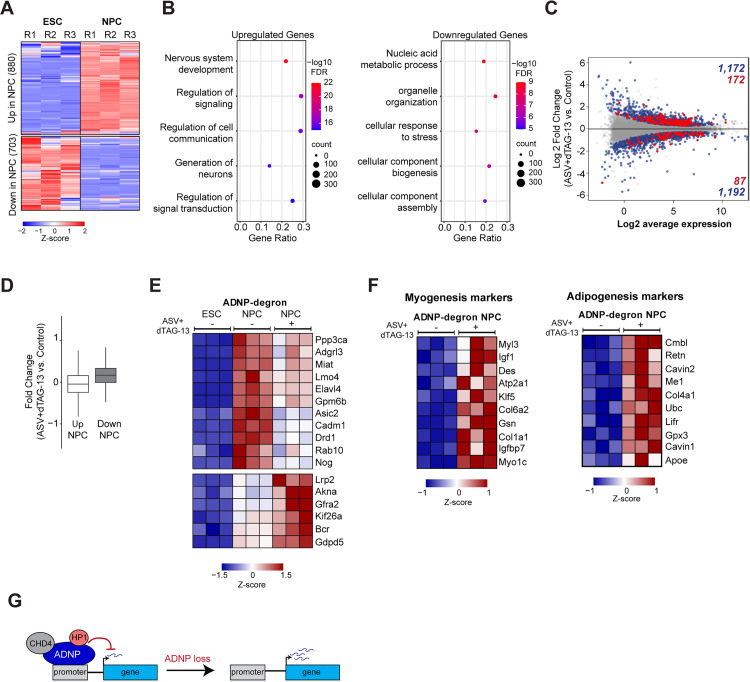
ADNP depletion in NPCs derepresses non-specific lineage markers. **(A)** Heatmap showing expression in control ADNP^DD^ mESCs and NPCs of 1,583 NPC-gained ADNP target genes that contain an NPC-gained ADNP peak across the promoter and/or gene body. Genes are clustered into 880 that are upregulated in NPCs (top) and 703 that are downregulated (bottom) and scaled by row Z-score. **(B)** Gene ontology of NPC-gained ADNP target genes that are upregulated (880 genes, left) or downregulated (703 genes, right) in NPCs. Adjusted p-value based on the FDR calculation from DAVID (Benjamini-Hochberg adaptive control for multiple testing). **(C)** MA plot of RNA-seq expression between control and drug-treated ADNP^DD^ NPCs. Blue dots indicate differentially expressed genes (adjusted P-value ≤ 0.05). Red dots indicate differentially expressed genes that contain an NPC-gained ADNP peak in the promoter and/or gene body. **(D)** Boxplot of log2 expression fold changes in drug-treated versus control ADNP^DD^ NPCs for NPC-gained ADNP target genes that are significantly upregulated (left) or downregulated (right) in NPCs compared to mESCs. Box, 25th percentile – median – 75th percentile. Whiskers extend to 1.5x interquartile range. **(E)** Heatmaps showing expression of select genes in control ADNP^DD^ mESCs, control ADNP^DD^ NPCs, and drug-treated ADNP^DD^ NPCs. Genes displayed are upregulated in NPCs compared to ESCs, contain an NPC-gained ADNP peak across their promoter, and are differentially expressed in drug-treated NPCs compared to control. Genes are scaled by row Z-score. **(F)** Heatmaps showing expression in control and drug-treated ADNP^DD^ NPCs of top 10 myogenesis and adipogenesis markers as identified by GSEA. Genes are scaled by row Z-score. **(G)** Model for CTCF-independent regulation of gene expression by ADNP. ADNP binding to gene promoters in NPCs repress gene expression through interactions with other proteins such as CHD4 and HP1. In the absence of ADNP, loss of repressive activity permits gene expression.

ADNP is gained at specific sites in NPCs and a large majority of these are over promoters or bodies of genes (Fig 2A). These sites are enriched for H3K27ac (Fig 2C) but contain low levels of CTCF (S1E Fig) and do not accumulate CTCF upon ADNP loss in NPCs (Fig 4A), suggesting that ADNP regulates expression of these “NPC-specific ADNP target genes” in a CTCF-independent manner. Using our RNA-seq data from control ADNP^DD^ mESCs and NPCs, we asked how the expression of 1,583 NPC-specific ADNP target genes change upon neurodifferentiation. We found that compared to expression in ESCs, 880 genes were upregulated and 703 genes were downregulated in NPCs (Fig 5A). Gene ontology (GO) analysis revealed that upregulated target genes were enriched in several processes related to neurodevelopment such as nervous system development, generation of neurons, and regulation of cell communication (Fig 5B, left). Consistent with decreased proliferative potential as mESCs differentiate into NPCs, genes involved in nucleic acid metabolism and cellular component biogenesis and assembly were downregulated (Fig 5B, right). Next, we asked how NPC-specific ADNP gene targets are affected by ADNP loss. Our differential expression analysis identified 1,172 upregulated and 1,192 downregulated genes (Fig 4H). Of these, 172 upregulated genes (14.7%) are NPC-gained ADNP targets, representing a 1.57-fold enrichment (p-value = 7.7 x 10^-10^, hypergeometric test), while only 87 downregulated genes (7.3%) are targets, a 1.28-fold under-enrichment (p-value = 0.0056, hypergeometric test) (Fig 5C).

We examined how NPC-gained ADNP targets that are upregulated versus downregulated in the mESC-to-NPC transition are impacted by ADNP depletion in NPCs. Upregulated genes were evenly split between further upregulation and loss of expression upon ADNP loss (Fig 5D, left). These upregulated ADNP targets are enriched for genes associated with neurodevelopment and nervous system function (Fig 5A and 5B). We subset genes identified in the “nervous system development” ontology (Fig 5B) to those containing NPC-gained ADNP over the promoter and found that they were similarly divided into both up- and downregulated groups upon ADNP depletion (Fig 5E), suggesting that ADNP can either promote or alternatively prevent over-expression of these neural genes.

In contrast to upregulated genes, ADNP target genes normally downregulated in NPCs became mostly derepressed (Fig 5D, right), consistent with a role for NPC-gained ADNP in transcriptional repression. We performed gene set enrichment analysis (GSEA) to identify pathways that were dysregulated upon ADNP depletion in NPCs. GSEA identified several non-neuronal hallmark gene sets that were upregulated in ADNP-depleted NPCs, including myogenesis and adipogenesis markers (Figs 5F, S5A, and S5B), indicating that loss of ADNP results in the derepression of genes normally involved in mesoderm differentiation. We further confirmed that *Ucp2* and *Myo1c,* genes with functions in adipogenesis and myogenesis respectively, contain ADNP at their promoters and are upregulated when ADNP is depleted (S5C Fig). Overall, our results indicate that ADNP-mediated gene regulation in NPCs guides neural lineage commitment while suppressing alternative cell fates, and that this occurs via CTCF-dependent (Fig 4J) as well as CTCF-independent mechanisms (Fig 5G).

## Discussion

In this study, we identify genome-wide shifts in ADNP distribution upon differentiation of mouse embryonic stem cells to neural progenitors, providing new insight into functions and potential mechanisms of ADNP in the neural lineage. We found that ADNP occupancy of many CTCF-enriched sites in mESCs is retained during differentiation to NPCs, but that ADNP also binds *de novo* to thousands of sites in NPCs that do not contain CTCF but are instead enriched for active promoters and H3K27ac (Figs 1 and 2). Using a novel degron model for acute ADNP depletion (Fig 3), we found that shared ADNP sites retain their role in regulation of CTCF genomic localization, as ADNP depletion in NPCs results in gained CTCF at these sites and changes in chromatin looping without significant alterations in genome compartmentalization or in topologically associated domains (Fig 4). In contrast, ADNP at NPC-specific promoter sites acts to calibrate the expression of neural lineage genes while transcriptionally repressing non-neural lineage genes in a CTCF-independent manner (Fig 5).

ADNP is lost, gained, or retained at distinct genomic sites when ESCs are differentiated to NPCs. We found that sites where ADNP is present in both mESCs and NPCs are also enriched for SINE B2 elements. ADNP was previously reported to bind SINE B2 retrotransposons in mESCs and regulate the genomic association of CTCF whose binding sites are enriched within these elements in the mouse genome [23]. Using our degron system for acute depletion of ADNP, we further demonstrate that loss of ADNP in differentiated NPCs results in accumulation of CTCF across these conserved ADNP sites. These CTCF gains promote the formation of short-range chromatin loops, often at the expense of long-range looping interactions, that often in turn promotes the aberrant upregulation of nearby genes (Fig 4). Some CTCF gains may alternatively result in new boundary formation that can insulate genes from enhancers and reduce rather than increase expression. While this appears less evident in our results, we do not rule out individual sites where this may occur. Interestingly, the functions of ADNP at *de novo* sites appear largely independent of CTCF as these sites do not gain CTCF upon ADNP depletion. These *de novo* ADNP sites are not enriched for SINE B2 retrotransposons and are instead enriched for MIR elements, which are known to harbor tissue specific enhancers [33,34]. ADNP was discovered in a previous study designed to identify protein factors that localize to active enhancers in mESCs [41]. It is possible that through its relocalization to MIR elements, ADNP retains its function at enhancers that become activated during nervous system development.

Despite its known function in transcriptional repression through its association with HP1 and CHD4 proteins, several lines of evidence point to a role for ADNP at active genes. ADNP localizes to active enhancers in mESCs [41] and to gene promoters that contain H3K27 acetylation in NPCs (Fig 2C). How ADNP localizes to these regions is not clear. As one possibility, ADNP was identified in proteomic screens for proteins that function at or proximal to R-loops, chromatin structures that form co-transcriptionally [28,42]. We also discovered that ADNP can resolve R-loop chromatin structures in vitro and in vivo [28], suggesting that ADNP may be able to recognize these and/or G quadruplex (G4) structures that frequently form on the displaced single strand of R-loops [43]. Supporting this premise, we observe that ADNP localizes to genes that become transcriptionally active in NPCs, thereby gaining R-loops and G4s, and is lost from genes that exhibit reduced expression and consequently lose R-loops in NPCs compared to ESCs. It is also possible that ADNP is directed to *de novo* regions in NPCs through its association with other transcription factors. Indeed, ADNP is known to associate with several chromatin remodelers in mESCs, including CHD4, BRG1, and ATRX, with context dependent roles in gene activation or repression [25,44]. Another open question is if and how ADNP may regulate H3K27Ac at active sites. It is possible that ADNP, through its chromatin remodeler associations, may limit H3K27Ac by restricting access of histone acetyltransferases to promoters and other active sites. Finally, identification of NPC-specific binding partners of ADNP may help reveal the existence of cell-type specific ADNP complexes with functions similar to or distinct from the ChAHP complex in mESCs and establish the principles of ADNP recruitment and function at specific genomic regions.

ADNP loss in mESCs results in upregulation of *Gata4* and *Sox17* genes, both important regulators of mesoderm and endoderm lineage differentiation [25]. We find that ADNP depletion in NPCs results in upregulation of myogenesis and adipogenesis pathways, both arising from cells of the mesoderm lineage. Our results thus support a role for ADNP in preventing the spurious expression of genes that are normally expressed in non-neural cells and therefore maintaining lineage fidelity during neurodevelopment. This finding may provide a mechanistic explanation for the neurodevelopmental disorders caused by *ADNP* mutations. ADNP also localizes to some genes that become expressed and have function in the neural lineage, some of which become downregulated upon ADNP depletion while others are instead further upregulated. Several neurodevelopmental disorders are caused by genetic mutations that result in insufficient expression of critical regulatory proteins including Rett Syndrome [45], ADNP syndrome [26], and CHD8-related neurodevelopmental disorder [46,47]. However, an abundance of these regulatory proteins can also be detrimental to normal development, as is seen in MECP2 duplication syndrome [48]. Thus, ADNP may function to ensure proper expression levels of neural lineage genes after they have been activated in response to specific developmental cues. Redistribution of ADNP during the ESC-to-NPC transition may also serve to provide temporal control to the differentiation process, for example by tapering the activation of immediate early genes such as *Fosl2* past a certain timepoint. In the absence of ADNP, such genes may continue to be improperly expressed throughout later steps of differentiation to a degree that impedes successful transition into the neuronal lineage.

It is also interesting that ADNP has a role in repressing mesoderm lineage genes when recent studies have reported a role for ADNP in microglia, specialized cells in the nervous system that function as immune regulators and that are derived from mesoderm progenitors [49]. Deletion of ADNP in microglia resulted in altered phagocytosis and interactions with neurons in coculture. Cellular analysis of ADNP in this cell type revealed that unlike mESCs and NPCs, ADNP was not localized to the nucleus in microglia and instead accumulated in endocytic vesicles. Much remains unknown about the developmental timing of these cellular shifts in ADNP, including what cell type-specific sites, genes, and repeat elements it may localize to as well as what cell type-specific binding factors it cooperates with. We have demonstrated that our ADNP^DD^ cell line expresses endogenous ADNP at near-normal levels and can be selectively depleted of ADNP at any desired timepoint, including well after differentiation from the mESC state. This degron system therefore represents a valuable tool for continuing to probe roles of ADNP in different lineages, particularly the consequences of its loss at various developmental timepoints on cell identity and function. Such future experiments will build a more complete picture of ADNP’s roles in regulating neural lineage commitment and may reveal vulnerable pathways impacted by its loss that can serve as therapeutic targets in ADNP-associated disorders.

## Methods

### Experimental model and subject details

#### Mouse ESC culture.

Mouse embryonic stem cells (mESCs) were cultured feeder-free on 0.1% gelatin coated plates in mESC medium consisting of DMEM, 15% fetal bovine serum (Gibco), 1x non-essential amino acids, 1X GlutaMAX (Gibco 35050), 25mM HEPES, 100U/ml Pen-Strep, 55μM 2-mercaptoethanol, 3μM glycogen synthase kinase (GSK) inhibitor (Millipore 361559), 1μM MEK1/2 inhibitor (Millipore 444966), and LIF (Sigma, ESGRO). Cell lines have not been authenticated by simple tandem repeat profiling.

### Method details

#### Generation of ADNP-FKBP, ADNP-SMASh, and ADNP^DD^ mESCs.

A guide RNA sequence targeting the C-terminal region of ADNP was inserted into PX459, a gift from Feng Zhang [50] (Addgene plasmid: 62988). To generate the donor plasmid for ADNP-FKBP-V5, gBlock gene fragments were synthesized (IDT) and inserted into BamHI and EcoRV sites in pcDNA3 using NEBuilder. Donor plasmid for ADNP-SMASh was generated via NEBuilder by combining PCR products from ADNP-FKBP-V5 donor plasmid and pUC19_Ring1b-Cterm-BlastR-SMASh-donor, a gift from the Bonasio lab. Donor plasmid for ADNP-SMASh-FKBP (ADNP^DD^) was generated via NEBuilder by combining PCR products from ADNP-FKBP-V5 and ADNP-SMASh donor plasmids. gBlock and oligo sequences used to generate plasmids are listed in S1 Table.

ADNP-FKBP-V5, ADNP-SMASh, and ADNP-SMASh-FKBP (ADNP^DD^) cell lines were generated by transfecting 1 µg ADNP guide RNA plasmid and 1.5 µg corresponding donor plasmid into E14 mESCs with Lipofectamine 3000. Cell lines were selected using 1 μg/mL puromycin (ADNP-FKBP-V5) or 10 μg/mL blasticidin (ADNP-SMASh and ADNP^DD^). Clones were confirmed by performing PCR on genomic DNA extracted by QuickExtract (Epicentre QE09050). Efficacy of the ADNP^DD^ line was confirmed by Western blot on cells grown in the presence of 2 uM asunaprevir and 500 nM dTAG-13 for various timepoints.

#### Immunostaining and imaging of mESCS.

100,000 mESCs were cytospun onto glass slides. Slides were incubated for 5 minutes in ice cold PBS followed by 7 minutes in ice cold CSKT buffer (100mM NaCl, 300mM sucrose, 10mM PIPES pH 6.8, 3mM MgCl2, 0.5% Triton), fixed in 4% paraformaldehyde solution at room temperature for 10 minutes, and stored in 70% ethanol at 4°C. For immunofluorescence experiments, ADNP or CTCF antibody (Cell Signaling 3418S) was diluted in blocking solution and each incubation was followed by three washes with PBST (0.1% Tween 20 in PBS) at room temperature, 2 minutes each. Before immunostaining, slides were dehydrated with increasing concentrations of ethanol (70%, 80%, 90%, and 100%) for 2 minutes each. After air drying the slides for 5 minutes, the cells were blocked in blocking solution (1% BSA, 0.1% Tween 20 in PBS) for 20 minutes at room temperature and incubated with ADNP or CTCF antibody for 1 hour at room temperature followed by goat anti-rabbit IgG Alexa 555 for ADNP (Invitrogen, A21429, 1:500) or chicken anti-rabbit IgG Alexa 488 for CTCF (Invitrogen, A21441, 1:1000) for 30 minutes at room temperature, before being mounted in Vectashield containing DAPI (Vector Labs H-1200).

#### Differentiation of mouse embryonic stem cells to neural progenitor cells.

Differentiation of ADNP^DD^ mESCs to NPCs was performed as previously described [51]. Briefly, mESCs were plated onto gelatin-coated wells of a 6-well plate (30,000 cells per well) in mESC medium and cultured for 2–3 hours to allow attachment to the plate. To induce differentiation, mESC medium was withdrawn and N2B27 medium (50% Neurobasal medium, 50% DMEM/F-12 medium, 1 mM sodium pyruvate, 0.1 mM non-essential amino acids, 2 mM L-Glutamine, 0.5% Pen-Strep, 55 µM beta-mercaptoethanol, 40 µG/mL bovine serum albumin, 1x N-2 supplement, 1x B-227 supplement) containing 10 ng/mL human basic fibroblast growth factor (bFGF, Gemini Bio #300–112 P) was added. Media was replaced with N2B27 medium containing bFGF at 24 and 48 h after induction. At 72, 96 h, 120h, and 144h after induction, media was replaced with N2B27 medium containing 500 nM smoothened agonist (SAG, Sigma #566661). Cells were imaged and/or harvested for Western blots and genomics experiments on day 7 after induction. For experiments involving pre-treatment of ADNP^DD^ mESCs, 2 µM asunaprevir (ASV) and 500 nM dTAG-13 were added to cultured mESC plates 24 hr before plating onto 6-well plates and re-added with every change of media during the differentiation protocol. For experiments involving treatment of differentiated ADNP^DD^ NPCs, 2 µM ASV and 500 nM dTAG-13 were added to wells alongside media replacements at the day 5 and day 6 timepoints.

#### ChIP-seq.

ChIP-seq was performed as previously described on 5–15 million cells per sample [52]. Crosslinked DNA for ChIP-seq was sheared via sonication to an average size of 200–400 bp on a Covaris ME220 instrument (10% duty cycle, 75 W peak incident power, 1000 cycles per burst, 6 min). Immunoprecipitation was performed with 500 ng of CTCF antibody (Cell Signaling 3418S) or 5 µg of ADNP antibody.

#### RNA-seq.

RNA was extracted from cells using Trizol (Invitrogen), subjected to Turbo DNase digestion (Ambion AM2238), rRNA-depleted using FastSelect -rRNA HMR (Qiagen), and converted to cDNA using Ultra II Directional RNA Library Prep Kit (NEB E7760).

#### Hi-C.

Hi-C was performed using the Arima Genomics Hi-C+ for High-Coverage Kit on 4 million cells per sample.

#### Library preparation and sequencing.

DNA samples from ChIP-Seq and cDNA samples from RNA-Seq were end-repaired using End-Repair Mix (Enzymatics), A-tailed using Klenow exonuclease minus (Enzymatics), purified using MinElute columns (Qiagen), and ligated to Illumina adapters (NEB #E7600) using T4 DNA ligase (Enzymatics). Size selection for fragments >150 bp was performed using AMPure XP magnetic beads (Beckman Coulter). Libraries were PCR amplified with dual index primers (NEB #E7600) using Q5 DNA polymerase (NEB #M0491) and purified with MinElute. Libraries for Hi-C samples were prepared using the NEBNext Ultra II DNA Library Prep Kit with modifications according to the Arima Genomics Hi-C Kit. Sequencing of ChIP-seq, RNA-seq, and Hi-C libraries was performed on a NextSeq 2000 instrument (Illumina) with 61x2 paired-end cycle setting.

### Quantification and statistical analysis

#### Statistical test and measurement details.

Statistical details of experiments, including tests used, significance p-values, and definitions of centers, dispersions, and precisions, are listed when relevant alongside text, figures, and figure legends.

#### ChIP-seq alignment and analysis.

ChIP-seq reads were mapped to the mm10 mouse genome using Bowtie2 version 2.4.4 [53] with default paired-end settings. Discordantly aligning reads and PCR read duplicates were removed from BAM files using Samtools [54]. BigWig tracks were generated using the bamCoverage function in deepTools version 3.5.1 [55] using the parameters “--binSize 5 --extendReads --normalizeUsing CPM” and “--blackListFileName” to remove reads overlapping ENCODE blacklist regions [56]. Peaks were called with MACS2 2.2.7.1 [57] using the parameters “-f BAMPE -g mm --keep-dup 1 -q 0.001”. Signal plots and heatmaps of ChIP-seq data were generated using the computeMatrix, plotProfile, and plotHeatmap functions in deepTools. Differential binding analysis of ChIP-seq data was performed in R version 3.6.0 using DiffBind version 2.14.0 [58]. Sites of differential occupancy were called using the edgeR method and an FDR cutoff of 0.05. Genomic annotation of peaks was performed in R version 4.2.1 using ChIPseeker version 1.34.1 [59]. For Venn diagrams of peak overlap (S1A-S1B Fig), consensus peaksets were defined using peaks identified in both replicates of a sample group, and overlaps of at least 1 bp were counted. To determine enrichment of peak overlaps with repeat elements (S2 Fig), repeat element locations were obtained from RepeatMasker tables from the UCSC Genome Browser and the number of peaks overlapping a given repeat family was counted. This overlap was compared to a background set composed of 100 permutations of the peakset using the ChIPseeker shuffle function, and the average log2 fold enrichment relative to background was reported.

#### RNA-seq alignment and analysis.

RNA-Seq reads were aligned to mm10 using STAR version 2.7.9a [60]. RSEM version 1.3.3 [61] was used to obtain estimated and TPM counts. Analysis of RNA-Seq data was performed in R using limma version 3.54.2 [62] and edgeR version 3.40.2 [63] using an expression cutoff as determined using the built-in edgeR function “filterByExpr”. NPC-gained ADNP targets were defined as genes with an associated NPC-gained ADNP target over the promoter and/or gene body as annotated by ChIPseeker. PCoA plots were generated using the plotMDS function in limma. Heatmaps of gene expression were generated using the pheatmap R package. Functional annotation of genes was performed with the DAVID browser-based tool [64] using Ensembl IDs as input. The adjusted p-value was obtained from the FDR value reported in DAVID, which is based on Benjamini-Hochberg adaptive control of the false discovery rate. Gene set enrichment analysis was performed with the GSEA tool.

#### Hi-C sequencing alignment and analysis.

Hi-C reads were combined between two independent replicates, then aligned to the mm10 genome using Juicer [65] with default settings and an annotation file based on the Arima restriction enzyme digest. Hi-C contact maps were generated using FAN-C version 0.9.27 [66] using Knight-Ruiz normalization. Eigenvector for A/B compartments was calculated using the “compartments” function in FAN-C. Chromatin loops were called using the HiCCUPS function in Juicer Tools on the CPU setting and default parameters. Topologically associated domains (TADs) were called using OnTAD version 1.4 [67] using 25kb resolution data and default parameters. Aggregate peak analysis (APA) matrices were generated using the APA function in Juicer Tools. Subtraction maps of APA matrices were generated by subtracting control from drug-treated NPC contact values for each individual pixel.

#### External data.

We obtained BigWig and FASTQ files for NPC H3K27Ac and NPC RAD21 from GSE252218 [68]. Gene expression counts for wildtype mESC and NPC RNA-Seq were obtained from GSE171401 [28].

## Supporting information

S1 Table gBlock and oligo DNA used in this study. (XLSX)

S1 Fig Relationship between ADNP and CTCF in ESC and NPCs stages. **(A)** Density plots of log2 mESC CTCF signal across 14,232 ADNP peaks called in mESCs (blue line) and log2 NPC CTCF signal across 5,883 ADNP peaks called in NPCs (red line). **(B)** Signal plot of CTCF ChIP-seq signal in mESC and NPC stages across NPC-lost, shared, and NPC-gained CTCF peaks. RPM, reads per million. **(C)** Signal plot of ADNP ChIP-seq signal in mESC and NPC stages across NPC-lost, shared, and NPC gained ADNP peaks. RPM, reads per million. **(D)** Signal plot of CTCF ChIP-seq signal in mESC and NPC stages across all called NPC CTCF peaks versus NPC-gained ADNP peaks. RPM, reads per million. **(E)** Heatmap of CTCF ChIP-Seq signal in mESC and NPC stages across NPC-lost (9,372), shared (7,105), and NPC-gained (2,802) ADNP peaks. Rows within each group are sorted in decreasing order by mean signal across all samples. RPM, reads per million. **(F)** Signal plot of ADNP ChIP-seq signal in mESC and NPC stages across all called NPC ADNP peaks versus NPC-gained CTCF peaks. RPM, reads per million. **(G)** Heatmap of ADNP ChIP-Seq signal in mESC and NPC stages across NPC-lost (18,907), shared (20,249), and NPC-gained (10,977) ADNP peaks. Rows within each group are sorted in decreasing order by mean signal across all samples. RPM, reads per million.
(TIF)

S2 Fig Repeat element enrichment of ADNP and CTCF in mESCs and NPCs. Log2 fold enrichment over background of overlap between SINE B1, SINE B2, LINE1, MIR, ERVL, and ERVK repeat families at **(A)** NPC-lost ADNP peaks, **(B)** shared ADNP peaks, **(C)** NPC-gained ADNP peaks, **(D)** NPC-lost CTCF peaks, **(E)** shared CTCF peaks, or **(F)** NPC-gained CTCF peaks. **(G)** Barchart showing proportion of NPC-lost, shared, and NPC-gained ADNP and CTCF peaks that overlap a MIR repeat element compared to a set of randomly permuted genomic regions. ***, p-value < 0.001 by permutation testing against 10,000 permutations of random genomic regions.
(TIF)

S3 Fig Development of the ADNP^DD^ system and characteristics of ADNP^DD^ differentiation. **(A)** Western blot for V5, ADNP, and actin in WT mESCs and two ADNP-FKBP-V5 mESC lines with or without dTAG-13 treatment. Antibodies are indicated on the right. **(B)** Top, schematic of the ADNP-SMASh system. In the absence of drug treatment, the SMASh tag is self-cleaved from ADNP by NS3 protease, resulting in generation of wildtype ADNP. Upon addition of asunaprevir (ASV), protease activity is inhibited and ADNP-SMASh is targeted for degradation. Bottom, Western blot for ADNP and actin in WT mESCs and two ADNP-SMASh mESC lines with or without ASV treatment. Asterisk indicates a residual ADNP-SMASh protein band. Antibodies are indicated on the right. **(C)** Western blot for ADNP, CTCF, and actin in ADNP^DD^ mESCs with or without ASV and dTAG-13 treatment. Antibodies are indicated on the right. **(D)** Immunostaining of ADNP^DD^ mESCs for CTCF (green) before (control) and after 24hr treatment with ASV and dTAG-13. **(E)** Principal coordinate analysis of RNA-Seq gene expression in parental (wildtype) and ADNP^DD^ mESCs and NPCs. **(F)** Bar charts showing expression of the *Oct4 (Pou5f1)* and *Nanog* genes in counts per million (CPM) in ADNP^DD^ mESCs and NPCs. Circles indicate individual biological replicates. Data are presented as mean values ± SEM. **(G)** Bar charts showing expression of the *Sox4* and *Nes* genes in counts per million (CPM) in ADNP^DD^ mESCs and NPCs. Circles indicate individual biological replicates. Data are presented as mean values ± SEM. **(H)** Gene ontology of genes that are upregulated in ADNP^DD^ NPCs compared to mESCs.
(TIF)

S4 Fig CTCF and genome organization changes in response to acute depletion of ADNP in NPCs. **(A)** Volcano plot showing log2 fold changes in CTCF binding between control and drug-treated ADNP^DD^ mESCs on the x-axis and -log10 FDR on the y-axis. 1,102 gained sites with increased CTCF occupancy (tan) are indicated passing the 0.05 FDR cutoff for significance. No lost CTCF sites are identified. **(B)** Bar chart displaying distribution of CTCF sites that are gained and lost in drug-treated ADNP^DD^ NPCs, compared to all NPC CTCF peaks, across promoter, gene body, and intergenic genomic features. **(C)** Volcano plot showing log2 fold changes in CTCF binding between control and drug-treated ADNP^DD^ NPCs on the x-axis and -log10 FDR on the y-axis. Differentially bound CTCF sites are highlighted in red. Differentially bound CTCF sites that overlap a shared ADNP peak are highlighted in blue. **(D)** Volcano plot showing log2 fold changes in CTCF binding between control and drug-treated ADNP^DD^ NPCs on the x-axis and -log10 FDR on the y-axis. Differentially bound CTCF sites are highlighted in red. Differentially bound CTCF sites that overlap an NPC-gained ADNP peak are highlighted in blue. **(E)** 500-kb resolution Hi-C map of chromosome 15, showing observed contacts in control (upper right triangle) and drug-treated (lower left triangle) ADNP^DD^ NPCs. Tracks for eigenvector values denoting A/B compartmentalization in control and drug-treated NPCs are displayed below the map. **(F)** Bar chart showing number of topologically associated domains (TADs) identified in control and drug-treated ADNP^DD^ NPCs. **(G)** Bar chart showing number of chromatin loops identified in control and drug-treated ADNP^DD^ NPCs. **(H)** Boxplot showing size (in kb) of chromatin loops identified in control and drug-treated ADNP^DD^ NPCs. Box, 25th percentile – median – 75th percentile. Whiskers extend to 1.5x interquartile range. **(I)** Left, genome browser view of a region of chromosome 4 showing called chromatin loops and CTCF ChIP-seq signal (reads per million, RPM) in control and drug-treated ADNP^DD^ NPCs as well as H3K27Ac ChIP-seq signal (RPM) in wildtype NPCs. Chromatin loops called in control ADNP^DD^ NPCs are shown as dashed grey lines and those called in drug- treated NPCs are in red. Regions with CTCF gains are labeled with asterisks (*). Right, bar chart showing expression of the *Nol9* gene in counts per million (CPM) in control and drug-treated ADNP^DD^ NPCs. Circles indicate individual biological replicates. Data are presented as mean values ± SEM. **(J)** Left, genome browser view of a region of chromosome 1 showing called chromatin loops and CTCF ChIP-seq signal (reads per million, RPM) in control and drug-treated ADNP^DD^ NPCs as well as H3K27Ac ChIP-seq signal (RPM) in wildtype NPCs. Chromatin loops called in control ADNP^DD^ NPCs are shown as dashed grey lines and those called in drug- treated NPCs are in red. Regions with CTCF gains are labeled with asterisks (*). Right, bar chart showing expression of the *Rgl1* gene in counts per million (CPM) in control and drug-treated ADNP^DD^ NPCs. Circles indicate individual biological replicates. Data are presented as mean values ± SEM.
(TIF)

S5 Fig Non-specific lineage genes are derepressed upon ADNP depletion in NPCs. **(A, B)** GSEA results showing enrichment of genes upregulated in drug-treated ADNP^DD^ NPCs for myogenesis and adipogenesis hallmark sets. **(C)** Genome browser view of the *Ucp2* and *Myo1c* genes showing ADNP ChIP signal (reads per million, RPM) in NPCs.
(TIF)
