# Supplementary figures and images for: ADNP regulates chromatin architecture and lineage fidelity during neural differentiation
Source: PLoS Genet. 2026 Apr 1;22(4):e1012081. doi: 10.1371/journal.pgen.1012081 (PMC13061322; doi:10.1371/journal.pgen.1012081)

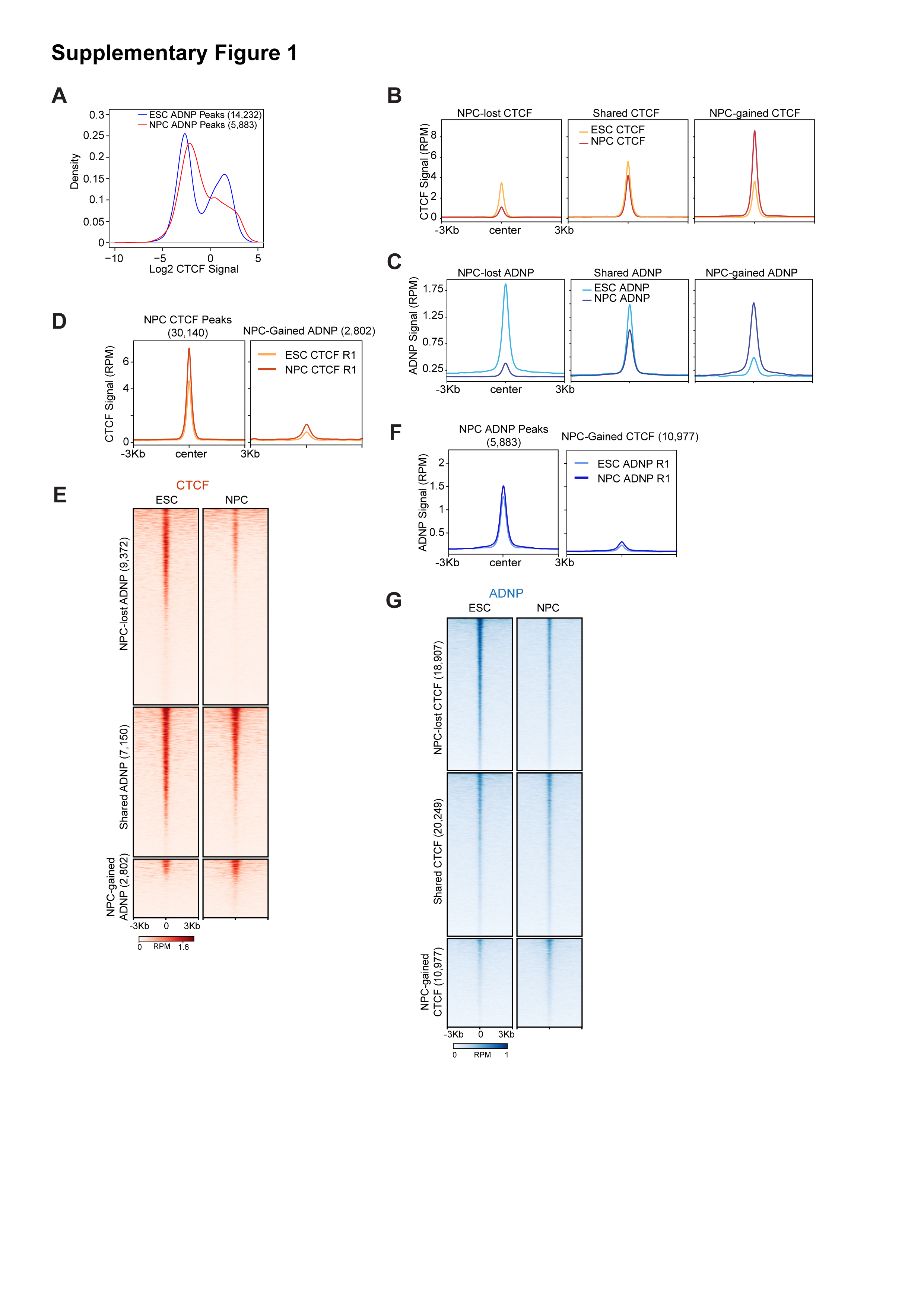

Supplement: S1 Fig — (A) Density plots of log2 mESC CTCF signal across 14,232 ADNP peaks called in mESCs (blue line) and log2 NPC CTCF signal across 5,883 ADNP peaks called in NPCs (red line). (B) Signal plot of CTCF ChIP-seq signal in mESC and NPC stages across NPC-lost, shared, and NPC-gained CTCF peaks. RPM, reads per million. (C) Signal plot of ADNP ChIP-seq signal in mESC and NPC stages across NPC-lost, shared, and NPC gained ADNP peaks. RPM, reads per million. (D) Signal plot of CTCF ChIP-seq signal in mESC and NPC stages across all called NPC CTCF peaks versus NPC-gained ADNP peaks. RPM, reads per million. (E) Heatmap of CTCF ChIP-Seq signal in mESC and NPC stages across NPC-lost (9,372), shared (7,105), and NPC-gained (2,802) ADNP peaks. Rows within each group are sorted in decreasing order by mean signal across all samples. RPM, reads per million. (F) Signal plot of ADNP ChIP-seq signal in mESC and NPC stages across all called NPC ADNP peaks versus NPC-gained CTCF peaks. RPM, reads per million. (G) Heatmap of ADNP ChIP-Seq signal in mESC and NPC stages across NPC-lost (18,907), shared (20,249), and NPC-gained (10,977) ADNP peaks. Rows within each group are sorted in decreasing order by mean signal across all samples. RPM, reads per million. (TIF) [file pgen.1012081.s002.tif]

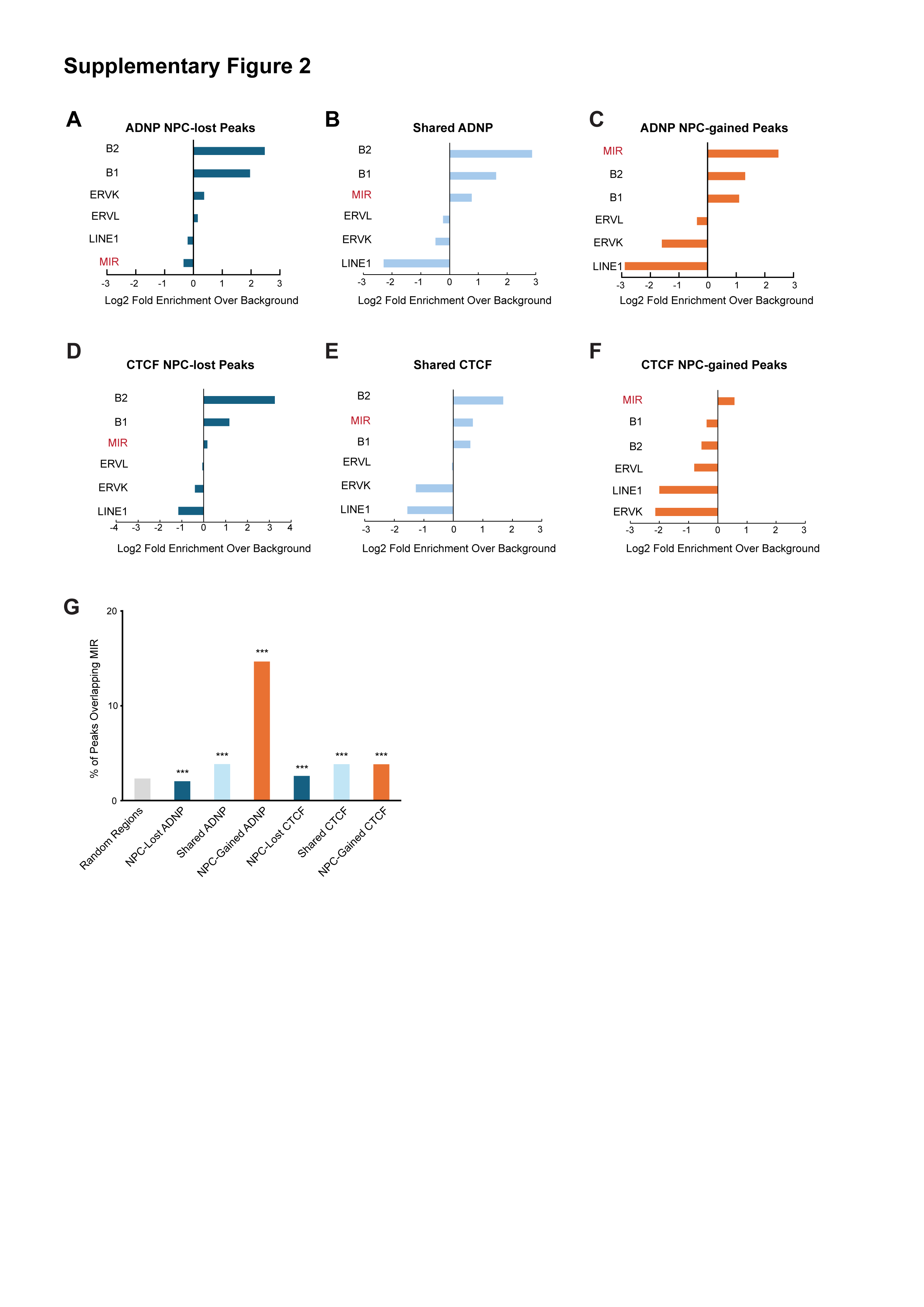

Supplement: S2 Fig — Log2 fold enrichment over background of overlap between SINE B1, SINE B2, LINE1, MIR, ERVL, and ERVK repeat families at (A) NPC-lost ADNP peaks, (B) shared ADNP peaks, (C) NPC-gained ADNP peaks, (D) NPC-lost CTCF peaks, (E) shared CTCF peaks, or (F) NPC-gained CTCF peaks. (G) Barchart showing proportion of NPC-lost, shared, and NPC-gained ADNP and CTCF peaks that overlap a MIR repeat element compared to a set of randomly permuted genomic regions. ***, p-value < 0.001 by permutation testing against 10,000 permutations of random genomic regions. (TIF) [file pgen.1012081.s003.tif]

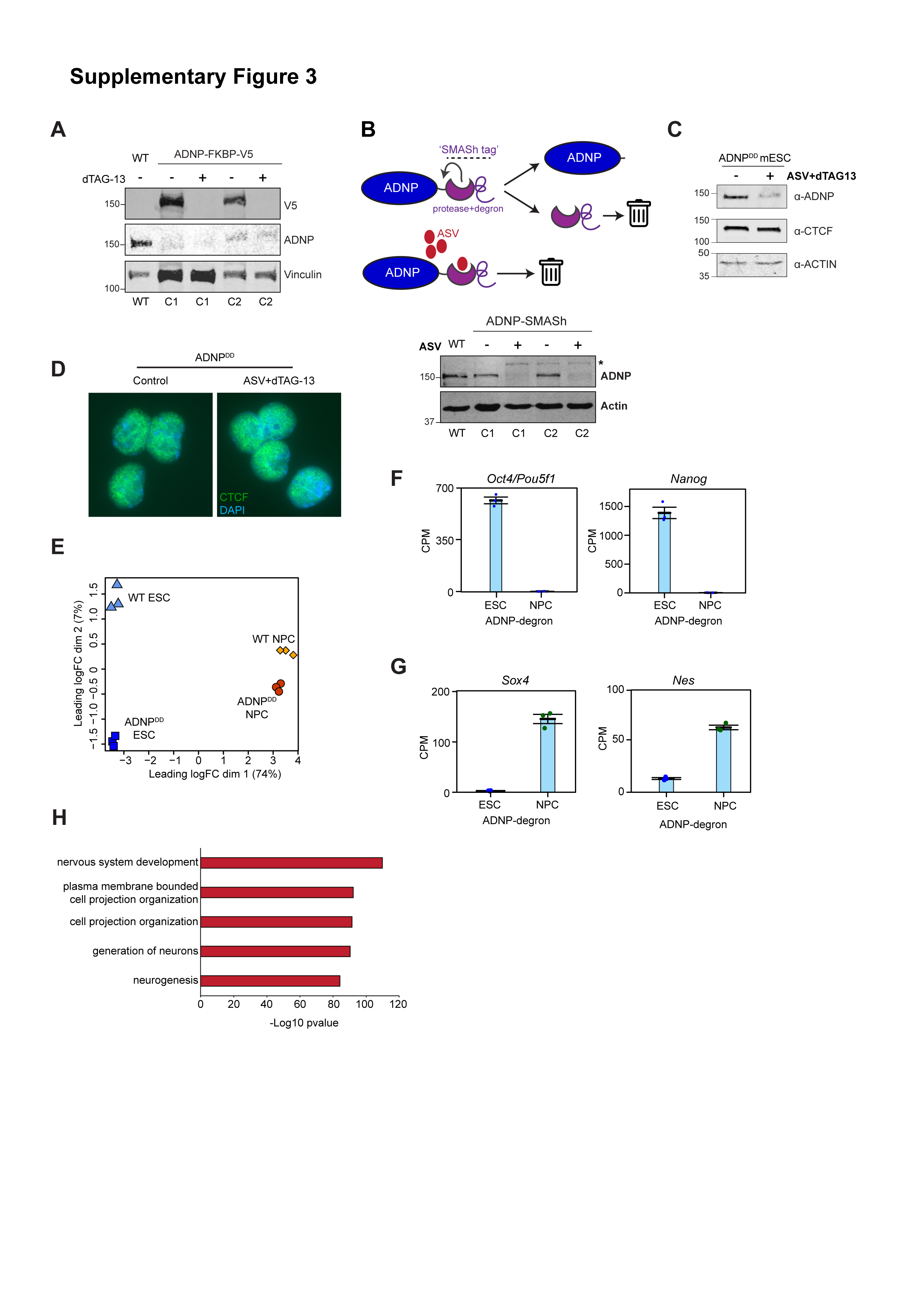

Supplement: S3 Fig — (A) Western blot for V5, ADNP, and actin in WT mESCs and two ADNP-FKBP-V5 mESC lines with or without dTAG-13 treatment. Antibodies are indicated on the right. (B) Top, schematic of the ADNP-SMASh system. In the absence of drug treatment, the SMASh tag is self-cleaved from ADNP by NS3 protease, resulting in generation of wildtype ADNP. Upon addition of asunaprevir (ASV), protease activity is inhibited and ADNP-SMASh is targeted for degradation. Bottom, Western blot for ADNP and actin in WT mESCs and two ADNP-SMASh mESC lines with or without ASV treatment. Asterisk indicates a residual ADNP-SMASh protein band. Antibodies are indicated on the right. (C) Western blot for ADNP, CTCF, and actin in ADNPDD mESCs with or without ASV and dTAG-13 treatment. Antibodies are indicated on the right. (D) Immunostaining of ADNPDD mESCs for CTCF (green) before (control) and after 24hr treatment with ASV and dTAG-13. (E) Principal coordinate analysis of RNA-Seq gene expression in parental (wildtype) and ADNPDD mESCs and NPCs. (F) Bar charts showing expression of the Oct4 (Pou5f1) and Nanog genes in counts per million (CPM) in ADNPDD mESCs and NPCs. Circles indicate individual biological replicates. Data are presented as mean values ± SEM. (G) Bar charts showing expression of the Sox4 and Nes genes in counts per million (CPM) in ADNPDD mESCs and NPCs. Circles indicate individual biological replicates. Data are presented as mean values ± SEM. (H) Gene ontology of genes that are upregulated in ADNPDD NPCs compared to mESCs. (TIF) [file pgen.1012081.s004.tif]

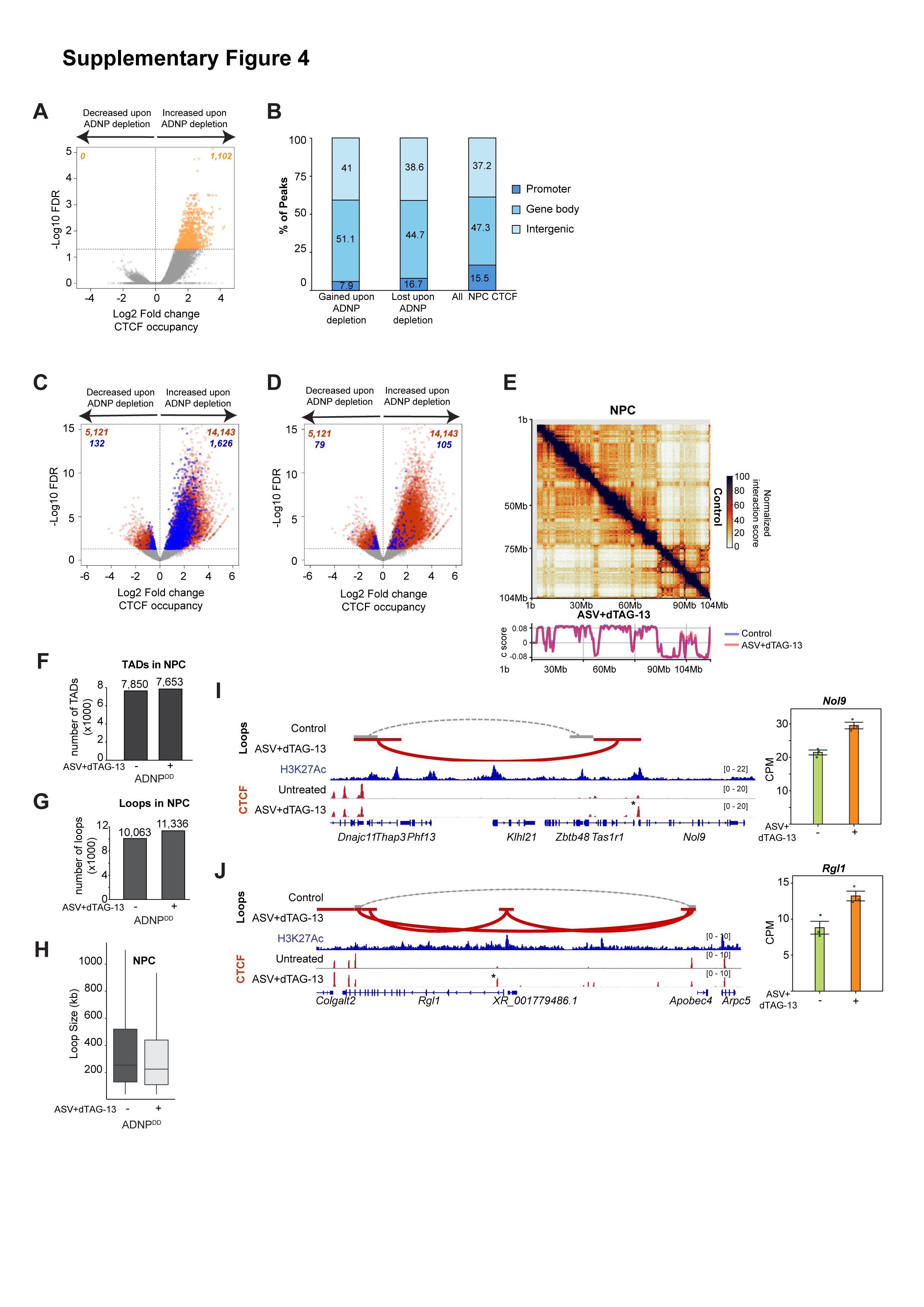

Supplement: S4 Fig — (A) Volcano plot showing log2 fold changes in CTCF binding between control and drug-treated ADNPDD mESCs on the x-axis and -log10 FDR on the y-axis. 1,102 gained sites with increased CTCF occupancy (tan) are indicated passing the 0.05 FDR cutoff for significance. No lost CTCF sites are identified. (B) Bar chart displaying distribution of CTCF sites that are gained and lost in drug-treated ADNPDD NPCs, compared to all NPC CTCF peaks, across promoter, gene body, and intergenic genomic features. (C) Volcano plot showing log2 fold changes in CTCF binding between control and drug-treated ADNPDD NPCs on the x-axis and -log10 FDR on the y-axis. Differentially bound CTCF sites are highlighted in red. Differentially bound CTCF sites that overlap a shared ADNP peak are highlighted in blue. (D) Volcano plot showing log2 fold changes in CTCF binding between control and drug-treated ADNPDD NPCs on the x-axis and -log10 FDR on the y-axis. Differentially bound CTCF sites are highlighted in red. Differentially bound CTCF sites that overlap an NPC-gained ADNP peak are highlighted in blue. (E) 500-kb resolution Hi-C map of chromosome 15, showing observed contacts in control (upper right triangle) and drug-treated (lower left triangle) ADNPDD NPCs. Tracks for eigenvector values denoting A/B compartmentalization in control and drug-treated NPCs are displayed below the map. (F) Bar chart showing number of topologically associated domains (TADs) identified in control and drug-treated ADNPDD NPCs. (G) Bar chart showing number of chromatin loops identified in control and drug-treated ADNPDD NPCs. (H) Boxplot showing size (in kb) of chromatin loops identified in control and drug-treated ADNPDD NPCs. Box, 25th percentile – median – 75th percentile. Whiskers extend to 1.5x interquartile range. (I) Left, genome browser view of a region of chromosome 4 showing called chromatin loops and CTCF ChIP-seq signal (reads per million, RPM) in control and drug-treated ADNPDD NPCs as well as H3K27Ac ChIP [file pgen.1012081.s005.tif]

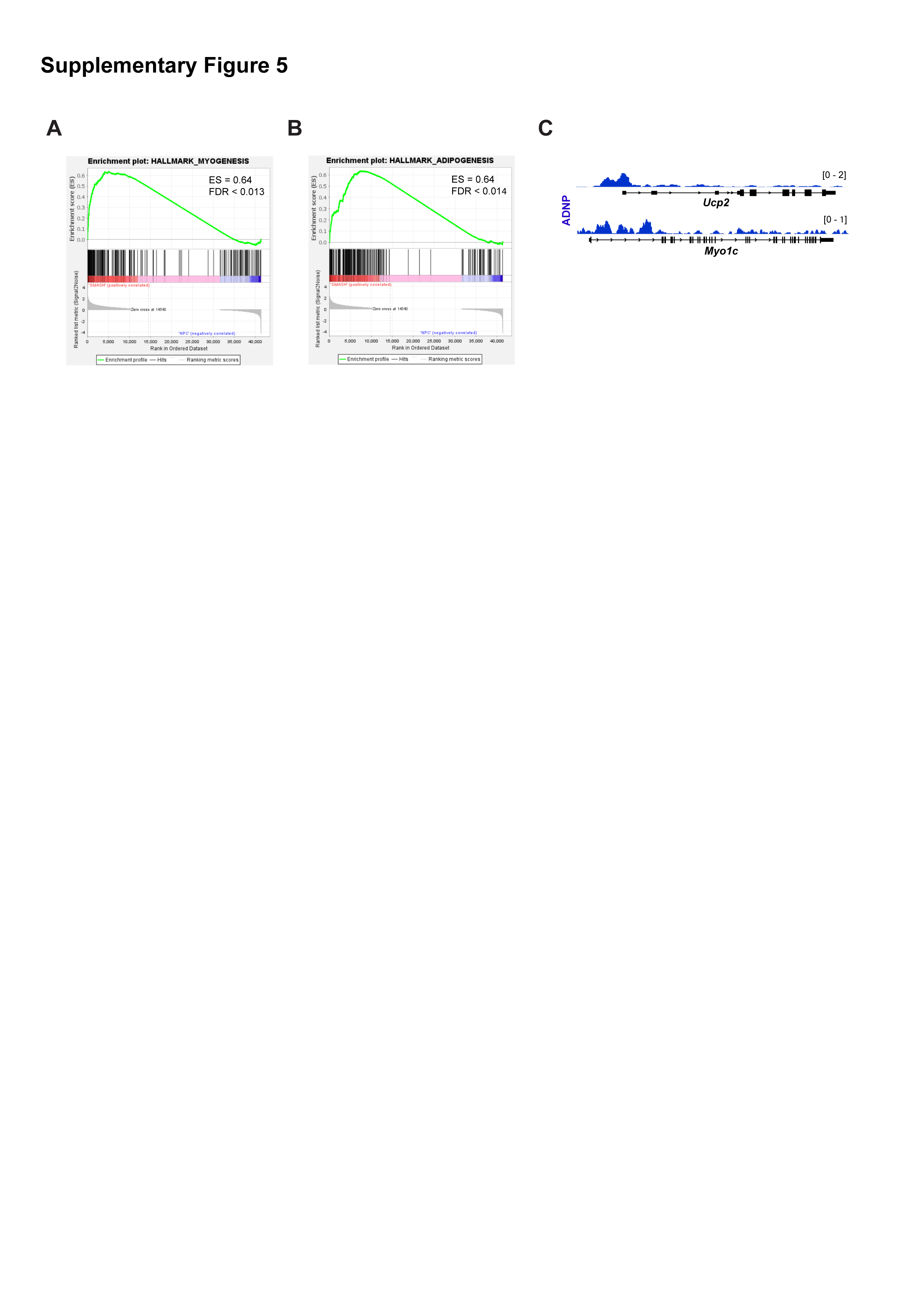

Supplement: S5 Fig — (A, B) GSEA results showing enrichment of genes upregulated in drug-treated ADNPDD NPCs for myogenesis and adipogenesis hallmark sets. (C) Genome browser view of the Ucp2 and Myo1c genes showing ADNP ChIP signal (reads per million, RPM) in NPCs. (TIF) [file pgen.1012081.s006.tif]
